# Supplementary material for: Eigenvector alignment: Assessing functional network changes in amnestic mild cognitive impairment and Alzheimer’s disease
Source: PLoS One. 2020 Aug 27;15(8):e0231294. doi: 10.1371/journal.pone.0231294 (PMC7451578; doi:10.1371/journal.pone.0231294)
Supplement: S1 Table — (PDF) [file pone.0231294.s001.pdf]

# S1 Table

| | |
|---|---|
| 1 | Frontal Pole Right |
| 2 | Frontal Pole Left |
| 3 | Insular cortex Right |
| 4 | Insular cortex Left |
| 5 | Superior Frontal gyrus Right |
| 6 | Superior Frontal gyrus Left |
| 7 | Middle Frontal gyrus Right |
| 8 | Middle Frontal gyrus Left |
| 9 | Inferior Frontal gyrus, pars triangularis Right |
| 10 | Inferior Frontal gyrus, pars triangularis Left |
| 11 | Inferior Frontal gyrus, pars opercularis Right |
| 12 | Inferior Frontal gyrus, pars opercularis Left |
| 13 | Precentral gyrus Right |
| 14 | Precentral gyrus Left |
| 15 | Temporal Pole Right |
| 16 | Temporal Pole Left |
| 17 | Superior Temporal gyrus, anterior division Right |
| 18 | Superior Temporal gyrus, anterior division Left |
| 19 | Superior Temporal gyrus, posterior division Right |
| 20 | Superior Temporal gyrus, posterior division Left |
| 21 | Middle Temporal gyrus, anterior division Right |
| 22 | Middle Temporal gyrus, anterior division Left |
| 23 | Middle Temporal gyrus, posterior division Right |
| 24 | Middle Temporal gyrus, posterior division Left |
| 25 | Middle Temporal gyrus, temporooccipital part Right |
| 26 | Middle Temporal gyrus, temporooccipital part Left |
| 27 | Inferior Temporal gyrus, anterior division Right |
| 28 | Inferior Temporal gyrus, anterior division Left |
| 29 | Inferior Temporal gyrus, posterior division Right |
| 30 | Inferior Temporal gyrus, posterior division Left |
| 31 | Inferior Temporal gyrus, temporooccipital part Right |
| 32 | Inferior Temporal gyrus, temporooccipital part Left |
| 33 | Postcentral gyrus Right |
| 34 | Postcentral gyrus Left |
| 35 | Superior Parietal Lobule Right |
| 36 | Superior Parietal Lobule Left |
| 37 | Supramarginal gyrus, anterior division Right |
| 38 | Supramarginal gyrus, anterior division Left |
| 39 | Supramarginal gyrus, posterior division Right |
| 40 | Supramarginal gyrus, posterior division Left |
| 41 | Angular gyrus Right |
| 42 | Angular gyrus Left |
| 43 | Lateral Occipital cortex, superior division Right |
| 44 | Lateral Occipital cortex, superior division Left |
| 45 | Lateral Occipital cortex, inferior division Right |
| 46 | Lateral Occipital cortex, inferior division Left |

**Table S1.1.** A list of the ROIs (ID: 1 – 47) identified according to the CONN atlas.

| | |
|---|---|
| 47 | Intracalcarine cortex Right |
| 48 | Intracalcarine cortex Left |
| 49 | Frontal Medial cortex |
| 50 | Juxtapositional Lobule cortex -formerly Supplementary Motor cortex- Right |
| 51 | Juxtapositional Lobule cortex -formerly Supplementary Motor cortex- Left |
| 52 | Subcallosal cortex |
| 53 | Paracingulate gyrus Right |
| 54 | Paracingulate gyrus Left |
| 55 | Cingulate gyrus, anterior division |
| 56 | Cingulate gyrus, posterior division |
| 57 | Precuneus cortex |
| 58 | Cuneal cortex Right |
| 59 | Cuneal cortex Left |
| 60 | Frontal Orbital cortex Right |
| 61 | Frontal Orbital cortex Left |
| 62 | Parahippocampal gyrus, anterior division Right |
| 63 | Parahippocampal gyrus, anterior division Left |
| 64 | Parahippocampal gyrus, posterior division Right |
| 65 | Parahippocampal gyrus, posterior division Left |
| 66 | Lingual gyrus Right |
| 67 | Lingual gyrus Left |
| 68 | Temporal Fusiform cortex, anterior division Right |
| 69 | Temporal Fusiform cortex, anterior division Left |
| 70 | Temporal Fusiform cortex, posterior division Right |
| 71 | Temporal Fusiform cortex, posterior division Left |
| 72 | Temporal Occipital Fusiform cortex Right |
| 73 | Temporal Occipital Fusiform cortex Left |
| 74 | Occipital Fusiform gyrus Right |
| 75 | Occipital Fusiform gyrus Left |
| 76 | Frontal Operculum cortex Right |
| 77 | Frontal Operculum cortex Left |
| 78 | Central Opercular cortex Right |
| 79 | Central Opercular cortex Left |
| 80 | Parietal Operculum cortex Right |
| 81 | Parietal Operculum cortex Left |
| 82 | Planum Polare Right |
| 83 | Planum Polare Left |
| 84 | Heschl's gyrus Right |
| 85 | Heschl's gyrus Left |
| 86 | Planum Temporale Right |
| 87 | Planum Temporale Left |
| 88 | Supracalcarine cortex Right |
| 89 | Supracalcarine cortex Left |

**Table S1.2.** A list of the ROIs (ID: 47 – 89) identified according to the CONN atlas.

| | |
|---|---|
| 90 | Occipital Pole Right |
| 91 | Occipital Pole Left |
| 92 | Thalamus Right |
| 93 | Thalamus Left |
| 94 | Caudate Right |
| 95 | Caudate Left |
| 96 | Putamen Right |
| 97 | Putamen Left |
| 98 | Pallidum Right |
| 99 | Pallidum Left |
| 100 | Hippocampus Right |
| 101 | Hippocampus Left |
| 102 | Amygdala Right |
| 103 | Amygdala Left |
| 104 | Accumbens Right |
| 105 | Accumbens Left |
| 106 | Brainstem |
| 107 | Cerebellum Crus1 Left |
| 108 | Cerebellum Crus1 Right |
| 109 | Cerebellum Crus2 Left |
| 110 | Cerebellum Crus2 Right |
| 111 | Cerebellum 3 Left |
| 112 | Cerebellum 3 Right |
| 113 | Cerebellum 4 5 Left |
| 114 | Cerebellum 4 5 Right |
| 115 | Cerebellum 6 Left |
| 116 | Cerebellum 6 Right |
| 117 | Cerebellum 7b Left |
| 118 | Cerebellum 7b Right |
| 119 | Cerebellum 8 Left |
| 120 | Cerebellum 8 Right |
| 121 | Cerebellum 9 Left |
| 122 | Cerebellum 9 Right |
| 123 | Cerebellum 10 Left |
| 124 | Cerebellum 10 Right |
| 125 | Vermis 1 2 |
| 126 | Vermis 3 |
| 127 | Vermis 4 5 |
| 128 | Vermis 6 |
| 129 | Vermis 7 |
| 130 | Vermis 8 |
| 131 | Vermis 9 |
| 132 | Vermis 10 |

**Table S1.3.** A list of the ROIs (ID: 90 – 132) identified according to the CONN atlas.
