# Supplementary material for: Eigenvector alignment: Assessing functional network changes in amnestic mild cognitive impairment and Alzheimer’s disease
Source: PLoS One. 2020 Aug 27;15(8):e0231294. doi: 10.1371/journal.pone.0231294 (PMC7451578; doi:10.1371/journal.pone.0231294)
Supplement: S2 Table — (PDF) [file pone.0231294.s002.pdf]

S2 Table

ID	1	2	3	ID	1	2	3	ID	1	2	3
1	8	4	10	47	1	4	3	93	4	5	6
2	5	2	2	48	1	4	6	94	2	2	0
3	6	4	6	49	4	4	4	95	4	10	1
4	13	3	2	50	9	5	7	96	2	4	3
5	6	3	13	51	11	4	4	97	2	3	3
6	5	0	7	52	6	3	2	98	3	1	0
7	2	4	3	53	8	5	2	99	5	21	9
8	5	5	3	54	13	9	2	100	2	2	8
9	5	3	1	55	0	3	5	101	2	7	13
10	4	2	7	56	1	4	1	102	2	8	8
11	4	4	5	57	1	2	4	103	5	1	3
12	4	6	11	58	2	9	6	104	4	19	4
13	4	4	4	59	2	6	1	105	7	16	4
14	2	3	2	60	11	7	4	106	5	6	7
15	4	4	7	61	9	3	7	107	0	9	7
16	4	1	7	62	7	4	5	108	4	3	2
17	2	1	3	63	16	4	8	109	7	1	6
18	3	3	3	64	2	21	11	110	2	11	19
19	9	3	3	65	1	4	3	111	2	1	2
20	4	9	4	66	7	9	3	112	6	1	3
21	6	2	6	67	4	3	6	113	8	3	2
22	7	2	8	68	8	3	12	114	2	7	4
23	5	19	11	69	7	3	6	115	6	6	13
24	2	12	15	70	6	9	16	116	4	8	6
25	1	4	0	71	4	7	10	117	1	4	1
26	2	1	2	72	6	7	6	118	3	6	4
27	5	3	6	73	4	6	10	119	3	6	7
28	3	7	3	74	1	4	9	120	2	4	5
29	7	3	15	75	1	6	5	121	3	3	9
30	5	13	22	76	3	6	5	122	4	1	8
31	3	7	19	77	3	4	7	123	10	11	8
32	5	9	8	78	3	4	5	124	2	4	6
33	4	3	6	79	5	10	12	125	3	1	6
34	6	3	3	80	0	7	3	126	1	1	2
35	5	1	1	81	3	4	10	127	5	2	1
36	5	2	2	82	15	4	14	128	8	9	13
37	2	4	1	83	12	10	15	129	3	7	6
38	1	3	4	84	9	4	9	130	5	7	3
39	0	5	2	85	21	4	8	131	3	3	3
40	2	1	6	86	8	7	9	132	0	9	1
41	5	3	2	87	6	3	8				
42	2	5	4	88	2	7	6				
43	2	1	2	89	9	5	5				
44	12	12	4	90	3	5	4				
45	2	3	7	91	2	8	16				
46	4	5	5	92	3	2	3				

Table S2.1. Eigenvector alignment where the number of significant changes in eigenvector alignment for each ROI with respect to other ROI are detailed for the comparisons (1) AD versus HC, (2) aMCI versus HC, (3) AD versus aMCI.
