# Supplementary material for: Eigenvector alignment: Assessing functional network changes in amnestic mild cognitive impairment and Alzheimer’s disease
Source: PLoS One. 2020 Aug 27;15(8):e0231294. doi: 10.1371/journal.pone.0231294 (PMC7451578; doi:10.1371/journal.pone.0231294)
Supplement: Attachment — Submitted filename: Response_to_reviewers.pdf [file pone.0231294.s003.pdf]

Response to Reviewers

Additional Requirements

1. Please ensure that your manuscript meets PLOS ONE's style requirements, including those for file naming. The PLOS ONE style templates can be found at http://www.plosone.org/attachments/PLOSOne_formatting_sample_main_body.pdf and http://www.plosone.org/attachments/PLOSOne_formatting_sample_title_authors_affiliations.pdf

File links do not appear to work, but followed guidance on PLOS ONE website.

2. Please ensure that you refer to Figure 2 in your text as, if accepted, production will need this reference to link the reader to the figure.

Figure 2 is now referred to in text.

3. Please include captions for your Supporting Information files at the end of your manuscript, and update any in-text citations to match accordingly. Please see our Supporting Information guidelines for more information: http://journals.plos.org/plosone/s/supporting-information.

Supporting information captions included.

Review Comments to the Author

Reviewer #1: I think the idea of the study is very meaningful and interesting. Research design is appropiated and described fully. Results and conclusions are presented clearly. However, it would be useful for the reader to receive more information regarding the participants (demographic data, educational level, socioeconomic level, cognitive profile, neuropsychiatric symptoms).

Added additional information on participants in the Dataset subsection:

"Probable AD diagnosis was defined by NINCDS-ADRDA consensus criteria [1], with a general cognitive evaluation made using Mini-Mental State Examination (MMSE). The mean MMSE score was 21.5 (SD 3.7) for the AD group, 25.8 (SD 2.3) for the MCI group and 29.3 (SD 0.67) for the HC group. The mean age of the AD group was 72.3 (SD 8.3), the mean of the MCI group was 70.7 (SD 7.1) and the mean of the HC group was 66.0 (SD 9.6). The mean education was 8.6 (SD 3.6) in the AD group, 11.1 (SD 3.5) in the MCI group and 14.5 (SD 3.0) in the HC group. There were 6 females in the AD group, 4 in the MCI group and 3 in the HC group. For additional details on the participants in the dataset, see [2]."

[1] G. McKhann, D. Drachman, M. Folstein, R. Katzman, D. Price, and E. M. Stadlan, 'Clinical diagnosis of Alzheimer's disease: report of the NINCDS-ADRDA Work Group under the auspices of Department of Health and Human Services Task Force on Alzheimer's Disease', Neurology, vol. 34, no. 7, pp. 939–944, Jul. 1984, doi: 10.1212/wnl.34.7.939.

[2] D. Mascali et al., 'Intrinsic Patterns of Coupling between Correlation and Amplitude of Low-Frequency fMRI Fluctuations Are Disrupted in Degenerative Dementia Mainly due to Functional Disconnection', PLOS ONE, vol. 10, no. 4, p. e0120988, Apr. 2015, doi:

10.1371/journal.pone.0120988.

Additional information on neuropsychiatric symptoms, specific cognitive symptomology and socioeconomic status was not available for inclusion.

Reviewer #2: The authors present a novel and potentially interesting approach to network analysis in functional connectivity analysis of neuroimaging data. However, the technique is applied only in a single very small dataset. There is a wealth of available open access datasets this method could be applied to. For example, the ADNI dataset would be particularly relevant for this paper. It is entirely reasonable to develop the dataset in a small test dataset, then go on to test the validity of the technique in another dataset.

I do hope the authors would consider doing this, given the potentially interesting findings. However, in its current form it would be rash to accept eigenvector allgnment as a reliable method for this kind of analysis.

We completely agree that there is a lot of potential for applying our methods to larger datasets to both validate the results presented in the paper and explore the reliability of these results as biomarkers for disease. This is work we aspire to complete following this publication and we have adjusted the conclusion to reflect these points and make the provisional nature of these findings clearer:

> "This analysis formed a proof of concept for eigenvector alignment, which demonstrates clear potential for wider application but would benefit from further analysis on larger datasets that can confirm the reliability of this method and its validity in identifying biomarkers of disease."

In addition to this clarification, we have made every effort to increase the accessibility of our work by making the eigenvector alignment algorithm, relevant scripts and processed dataset available at [3]. In this way, we provide anyone with the tools to validate our work in this paper and investigate the performance on other datasets.

[3] Clark, R. (2020, June 10). Eigenvector Alignment (Version v1.0). Zenodo. http://doi.org/10.5281/zenodo.3888075

In addition, the authors need to demonstrate the the CDI is better than some of the other methods outlined in the introduction for detecting communities. One issue with community detection is the variability depending on choice of parameters (eg thresholding) and stochastic effects. The authors could consider if they can conclusively demonstrate the superiority of CDI in this respect.

Firstly, CDI is selected as it forms communities around the most influential network nodes, which creates an explicit link between the defined communities and the influence of nodes in the network. This link allows communities to be categorised as most or least influential and, hence, without this link the community size comparison would not be possible. We have attempted to clarify this point throughout the paper:

Text added in the Communities of dynamical influence section:

> “While many other community detection algorithms exist, CDI is the focus here as it explicitly connects network influence with community designation. It is also worth noting that, unlike many other community detection methods, CDI is deterministic and not susceptible to stochastic processes changing community designations.

Text added in the Discussion section:

> “The use of CDI for this analysis, rather than one of the plethora of other community detection methods, is justified through its explicit linkage of network division with global network influence, which is also fundamental to eigenvector alignment. Furthermore, it is worth highlighting that CDI is deterministic with no stochastic processes that are commonly found in community detection algorithms but can reduce the reliability of findings, such as those presented herein on community size.”

The reviewer has raised valid concerns around the issues of stochastic effects, thresholding, and other factors affecting the community designation. We have attempted to address these concerns by first noting that CDI is deterministic as is now highlighted in the previously quoted text. However, thresholding and other factors can influence CDI’s community designations. Hence, we have made efforts to add further analysis and discussion to support the findings of differences in community size. The additional analysis includes an extension to the influential community size comparison, where significant differences in the mean sizes of the two least and the two most influential communities are found. The analysis on the use of arbitrary thresholds, instead of the Cluster Span Threshold, has also been updated with significant results seen for certain threshold values and the community size relationship between groups remaining consistent:

> “The pattern of AD subjects having larger most influential and smaller least influential communities when compared with HC subjects is resilient to threshold variation.”

The CDI algorithm settings that could influence community designation are now highlighted in the text as the number of input eigenvectors and the choice of eigenvector scaling. Both of these aspects are analysed and their impact on the significance of the findings highlighted, while the community size pattern continues to be resilient to variation this time from the algorithm settings.

Finally, this analysis is replicable with the code used to identify communities of dynamical influence made available and referenced in the paper at [4].

[4] Clark R., Communities of Dynamical Influence (Version v1.0); 2020. Available from:https://doi.org/10.5281/zenodo.3878931
