# Supplementary material for: Eigenvector alignment: Assessing functional network changes in amnestic mild cognitive impairment and Alzheimer’s disease
Source: PLoS One. 2020 Aug 27;15(8):e0231294. doi: 10.1371/journal.pone.0231294 (PMC7451578; doi:10.1371/journal.pone.0231294)
Supplement: Attachment — Submitted filename: Response_to_reviewers.pdf [file pone.0231294.s004.pdf]

Comments to the Author

6. Review Comments to the Author

Please use the space provided to explain your answers to the questions above. You may also include additional comments for the author, including concerns about dual publication, research ethics, or publication ethics. (Please upload your review as an attachment if it exceeds 20,000 characters)

Reviewer #1: (No Response)

Reviewer #2: Many thanks for revising the manuscript, and in particular strengthening the methodological aspects of the paper. This is very much welcome. I appreciate the discussion of stochastic vs deterministic effect, this is much clearer.

I didn't perhaps explain my comments as well as I could have done previously. It's not clear to me whether this paper is 1. trying to demonstrate that this method can be applied in brain imaging, which is fairly trivial, 2. validating the method by reproducing a well known change found by another method, or, 3. proposing a new insight in to Alzheimer's disease. To help this, it would be useful to more clearly state a hypothesis to test.

The results as presented are therefore rather exploratory and a challenging to relate to what we know about AD. For example, Heschl's gyrus is not typically involved early in AD, and alignment of temporal lobe regions to the brainstem is difficult to explain. It is difficult to tell whether these hard to explain findings are an issue with the underlying method, the small sample size, or whether they represent a novel insight in to AD.

In order to assess the underlying method, it would be helpful to have a more clear hypothesis of expected findings based on previous work, or to compare with another method. I accept that comparing with other methods can be difficult when trying to demonstrate some superiority - ideally you'd like to show some overlap in the findings, but that the 'new' method demonstrates additional changes.

In order to clarify whether the small sample size is an issue, validating in a different dataset would be ideal. In many ways, it would be better to 'train' on the larger dataset and 'validate' on a smaller dataset. That's why I suggested ADNI which is readily available and has comparable data with functional imaging in AD, MCI and health control groups.

If the authors are trying to validate the method in neuroimaging in general, it would be better to choose to test an hypothesis that is more established rather than AD where there are conflicting reports, or to use modelled data such as with the virtual brain (https://www.thevirtualbrain.org/tvb/zwei).

Thank you for raising these concerns, I think in addressing your concerns we have significantly improved the clarity of the method and the reliability of the results.

In response to what the paper is trying to achieve we believe it now fulfils all 3 of your criteria:

- We believe it achieves 1 that as you state is fairly trivial.
- We agree that it did not convincingly achieve 2 in the previous submission and have now introduced a comparison between subject groups and random models, which validates that

eigenvector alignment functions as we'd expect with respect to the healthy control group in this dataset.

- We continue to propose insights that are not strongly associated with the AD pathology but have included further evidence from other studies that support these findings.

The issues highlighted with the paper appear to stem from a lack of clarity on how the underlying method functions and whether it produces reliable findings, especially on a small sample size. Starting with the underlying method, we have added a toy example in the methods section to illustrate that eigenvector alignment is essentially a comparison of pairwise functional connectivity. This toy example also highlights how our method's holistic assessment of the changing relationship between two ROIs differs from just assessing differences in functional connectivity.

Taking the intuitions gained from the toy example, we have added a validation of eigenvector alignment on the current dataset by comparing each subject group with random models of connectivity matrices. The results of these comparisons consistently display increased alignment for ROI pairs from either side of the same cortical area. This is as expected, where the same cortical area would be in closer alignment in subject data than in randomised networks. The use of different sets of random models shows that eigenvector alignment is capable of consistent detection of significant ROI pairs.

Following on from this validation step, the random model analysis is also used to filter the results in the subject group comparisons. In this way, only ROI pairs that display significantly different alignment from the sets of random models are eligible for comparisons between subject groups. This filter does influence the results of the paper but not enough to affect the main findings of the paper and adds reassurance to the reader that we have mitigated against false detections of significance given our sample size.

Finally, we have added additional discussion around the changes seen in the brainstem and auditory cortex by highlighting how these correspond with other research on AD and memory impairment. Including:

"A recent investigation using electrophysiological methods indicated hypersensitivity within the brainstem in people with MCI, which may indicate a compensatory process [38]."

"Functional connectivity changes in the Heschl's gyrus are associated with age-related hearing loss [40], which is a known risk factor for Alzheimer's disease [41]."
